# RNA-seq transcriptome profiling of porcine lung from two pig breeds in response to *Mycoplasma hyopneumoniae* infection

**DOI:** 10.7717/peerj.7900

**Published:** 2019-10-21

**Authors:** Ligang Ni, Chengyi Song, Xinsheng Wu, Xuting Zhao, Xiaoyan Wang, Bichun Li, Yuan Gan

**Affiliations:** 1 College of Animal Science and Technology, Yangzhou University, Yangzhou, Jiangsu, China; 2 Department of Animal Science and Technology, Jiangsu Agri-animal Husbandry Vocational College, Taizhou, Jiangsu, China; 3 Institute of Veterinary Medicine, Jiangsu Academy of Agricultural Sciences, Nanjing, Jiangsu, China

**Keywords:** RNA-seq, Jiangquhai pig, Duroc pig, Mycoplasma hyopneumoniae, Candidate gene

## Abstract

**Background:**

*Mycoplasma hyopneumoniae* (Mhp) is the main pathogen causing respiratory disease in the swine industry. Mhp infection rates differ across pig breeds, with Chinese native pig breeds that exhibit high fecundity (e.g., Jiangquhai, Meishan, Erhualian) more sensitive than Duroc, Landrace, and other imported pig breeds. However, the genetic basis of the immune response to Mhp infection in different pig breeds is largely unknown.

**Aims:**

The aims of this study were to determine the relative Mhp susceptibility of the Chinese native Jiangquhai breed compared to the Duroc breed, and identify molecular mechanisms of differentially expressed genes (DEGs) using an RNA-sequencing (RNA-seq) approach.

**Methods:**

Jiangquhai and Duroc pigs were artificially infected with the same Mhp dose. The entire experiment lasted 28 days. Daily weight gain, Mhp-specific antibody levels, and lung lesion scores were measured to evaluate the Mhp infection susceptibility of different breeds. Experimental pigs were slaughtered on the 28th day. Lung tissues were collected for total RNA extraction. RNA-seq was performed to identify DEGs, which were enriched by gene ontology (GO) and the Kyoto Encyclopedia annotation of Genes and Genomes (KEGG) databases. DEGs were validated with real-time quantitative polymerase chain reaction (RT-qPCR).

**Results:**

Infection with the same Mhp dose produced a more serious condition in Jiangquhai pigs than in Duroc pigs. Jiangquhai pigs showed poorer growth, higher Mhp antibody levels, and more serious lung lesions compared with Duroc pigs. RNA-seq identified 2,250 and 3,526 DEGs in lung tissue from Jiangquhai and Duroc pigs, respectively. The two breeds shared 1,669 DEGs, which were involved in immune-relevant pathways including cytokine-cytokine receptor interaction, PI3K-Akt signaling pathway, and chemokine signaling pathway. Compared to Jiangquhai pigs, more chemokines, interferon response factors, and interleukins were specifically activated in Duroc pigs; *CXCL10*, *CCL4*, *IL6* and *IFNG* genes were significantly up-regulated, which may help Duroc pigs enhance immune response and reduce Mhp susceptibility.

**Conclusion:**

This study demonstrated differential immune-related DEGs in lung tissue from the two breeds, and revealed an important role of genetics in the immune response to Mhp infection. The biological functions of these important DEGs should be further confirmed and maybe applied as molecular markers that improve pig health.

## Introduction

*Mycoplasma hyopneumoniae* (Mhp) exists in every country where pigs are raised and is the main pathogen leading to respiratory disease in the swine industry (Maes et al., 2008; Stark, Nicolet & Frey, 1998). The pathogen resides in the respiratory tract, and its secretions can be found in infected pigs for a long time (Maes et al., 1996). The main clinical symptoms of infected pigs are dry cough, as well as dramatically reduced porcine growth and feed conversion rates, which cause great losses to the pig industry (Maes et al., 1996; Sarradell et al., 2003).

Production practices on some Chinese pig farms revealed that Chinese local breeds are more sensitive to Mhp than imported breeds such as Duroc and Landrace. The Meishan and Erhualian, which are characterized by high fecundity, show extremely high susceptibility to Mhp infection (Fang et al., 2015; Maingi et al., 2014). This suggests that genetic components contribute to breed susceptibility or resistance to Mhp infection. Recently, it was reported that quantitative trait loci (QTLs) are associated with respiratory disease lesions, and five QTL were detected in Landrace pigs (Okamura et al., 2012). In Chinese Erhualian pigs, QTLs affecting respiratory disease were identified by a genome-wide association study; *CXCL6*, *CXCL8*, *KIT*, and *CTBP2* were highlighted as candidates that might associated with resistance or susceptibility to swine enzootic pneumonia-like respiratory disease (Huang et al., 2016). However, the genetic basis for the immune response to Mhp infection among different pig breeds remains largely unknown.

Jiangquhai is a Chinese pig breed distributed in the central Jiangsu Province. Similar to the Meishan and Erhualian breeds, Jiangquhai pigs exhibit sensitivity to Mhp infection (Maingi et al., 2014). Vaccines and antibiotics are used to control the occurrence of mycoplasma pneumonia of swine (MPS); however, these methods are not sufficient. It is therefore necessary to study the molecular mechanism of pathogenesis. This knowledge can be applied to carry out disease resistance breeding. In this study, we investigated the immune response of Jiangquhai and Duroc pigs to artificial Mhp infection using an RNA-sequencing (RNA-seq) approach. The goals were to identify genetic components that contribute to Mhp susceptibility or resistance and lay a foundation for genetic breeding that improve pig health.

## Materials & Methods

### Experimental design and sample collection

Twenty healthy 50-day-old Jiangquhai pigs were selected from the Jiangquhai Pig Breed Conservation Farm (Taizhou, China), and twenty healthy 50-day-old Duroc pigs were selected from the Xingtai Agriculture and Animal Husbandry Technology Development Company (Yangzhou, China). All pigs were free of all major porcine diseases and confirmed to be negative for Mhp, PRRSV, pseudorabies virus, and classical swine fever virus infection by PCR or reverse-transcription (RT)-PCR. Jiangquhai and Duroc pigs were randomly assigned to the control or infected group during the experiment and raised separately in isolation. Ten Jiangquhai and ten Duroc pigs were inoculated with 5 mL viral suspension of a virulent strain of Mhp (106 colour changing units [CCU]) (Xiong et al., 2014), which was provided by the Veterinary Medicine Institute of Jiangsu Academy of Agricultural Sciences (Nanjing, China). The remaining ten Jiangquhai and ten Duroc pigs were treated with an equivalent volume of aseptic physiological saline, which served as a negative control group. Four groups (i.e., Jiangquhai infection, Jiangquhai control, Duroc infection, Duroc control) were raised in isolation rooms to prevent cross-infection. Approval for the study was provided by the ethics committee of Yangzhou University (SYXK(Su) IACUC 2016-0131).

On day 28, all pigs were euthanized by stunning, and lung tissues were collected and stored at −70 °C. At this time, pulmonary MPS lesions were confirmed and assessed with the scoring system (Steinmann, Blaha & Meemken, 2014; Lee et al., 2011). From the beginning to the end (day 28) of the study, all experimental pigs were weighed prior to feeding in the morning to compare weight gain between groups. To assess the immune response, blood samples were collected on days 0 and 28 via jugular venipuncture into normal serum tubes without anticoagulant. The serum was separated by centrifugation (1,600× g for 10 min at 4 °C), divided into aliquots, and stored at −20 °C until analysis. Mhp-specific antibody in peripheral blood was detected.

### RNA-seq library preparation and sequencing

Total RNA was extracted from lung tissue of three infected and three control pigs of each breed (three pigs selected randomly from each group) using TRIzol reagent (Invitrogen, South San Francisco, CA, USA) following the manufacturer’s protocol. RNA integrity was evaluated using the Agilent 2100 Bioanalyzer (Agilent Technologies, Santa Clara, CA, USA). Samples with an RNA Integrity Number (RIN) >7 were subjected to subsequent analysis. The libraries were constructed using the TruSeq Stranded mRNA LTSample Prep kit (Illumina, San Diego, CA, USA) according to the manufacturer’s instructions. Then, these libraries were sequenced on the Illumina Hiseq 2500 platform (Shanghai OE Biotech Co., Ltd, Shanghai, China), and 125-bp paired-end reads were generated.

### Quality control and mapping

Raw data (raw reads) were processed using the NGS QC Toolkit (Ravi, Mukesh & Liu, 2012). Low-quality reads and those containing poly-N were removed to ensure high-quality mapping. Then the clean reads were mapped using the *Sus scrofa* genome 11.1 as a reference with bowtie2 (Langmead & Salzberg, 2012) or Tophat software packages (http://tophat.cbcb.umd.edu/) (Kim et al., 2013).

### RNA-seq data analysis

The FPKM (Fragments Per Kilobase of transcript per Million fragments mapped) value of each gene was calculated using cufflinks (Cole et al., 2012), and the read counts of each gene were obtained by HTSeq-Count (Anders, Pyl & Huber, 2015). Differentially expressed genes (DEGs) were identified using the DESeq estimateSizeFactors and nbinomTest functions, corrected *p*-value of 0.05 and log_2_ (Fold change) (log_2_ FC) of 1 were set as the threshold for significantly differential expression. Hierarchical clustering analysis of DEGs was performed to inspect sample relations. The DEGs were annotated by GO functional enrichment and KEGG pathway analysis using the Database for Annotation, Visualization and Integrated Discovery (DAVID).

### RT-qPCR verification

RNA was extracted from lung tissue of six infected and six control pigs of each breed using the TRIzol reagent following the manufacturer’s protocol. First-strand cDNA synthesis was performed using 5 µg of RNA and the Superscript II cDNA amplification system (Invitrogen, South San Francisco, CA, USA) according to the manufacturer’s protocol. Quantitative PCR was performed using an ABI 7500 real-time PCR system (Applied Biosystems, Foster City, CA, USA) and Power SYBR Green PCR Master Mix (Invitrogen, South San Francisco, CA, USA). The gene for glyceraldehyde 3-phosphate dehydrogenase (*GAPDH*) was included as an endogenous control, and the specific primers used in the RT-qPCR assays are listed in Table S1. Relative expression of target genes was determined by the comparative cycle threshold (C_T_) method (Livak & Schmittgen, 2001) and the ΔC_T_ value was calculated by subtracting the target C_T_ of each sample from its *GAPDH* C_T_ value.

### Statistical analysis

Weight gain and antibody level data are presented as mean ± standard error (SE). Comparison of variables was performed using one-way analysis of variance with SPSS13.0 software (SPSS Inc., Chicago, IL, USA).

## Results

### Effect of Mhp infection on weight gain, antibody production, and lung lesions in Jiangquhai and Duroc pigs

The average daily weight gain (ADG) of the Jiangquhai infected pigs was highly significantly lower than the Jiangquhai control pigs (*p* < 0.01), while the ADG of Duroc infected pigs was not significantly different from Duroc control pigs (*p* > 0.05) (Table 1). These results demonstrate that Mhp infection had a greater impact on the growth rate of Jiangquhai pigs compared to Duroc pigs.

**Table 1:** Average daily weight gain and Mhp-specific antibody levels of experimental pigs.

**Groups**	**N**	**Average body weight (kg)**		**ADG (g/d)**	**Antibody levels (s/p)**	
		**0d**	**28d**		**0d**	**28d**
Jiangquhai infection	10	8.54 ± 0.77	12.26 ± 0.51	132.86 ± 14.56^A^	0.05 ± 0.03	0.85 ± 0.20^A^
Jiangquhai control	10	8.46 ± 0.50	15.29 ± 0.76	244.11 ± 21.10^B^	0.05 ± 0.04	0.08 ± 0.07
Duroc infection	10	10.64 ± 0.83	18.41 ± 0.49	277.50 ± 25.81	0.02 ± 0.01	0.48 ± 0.19^B^
Duroc control	10	10.53 ± 0.72	18.81 ± 0.58	295.89 ± 18.86	0.02 ± 0.01	0.04 ± 0.01

**Notes.**

Different letters in the same column indicate significant differences of mean values (A and B) (*p* < 0.01).

On day 28, the level of Mhp-specific antibody (reported as the sample mean/positive control mean (s/p) value) in the peripheral blood of Jiangquhai infected pigs reached at 0.85 ± 0.20, which was significantly higher than that of Duroc infected pigs (0.48 ± 0.19) (*p* <  0.01) (Table 1). Mhp-specific antibody was not detected in control pigs.

**Figure 1:**
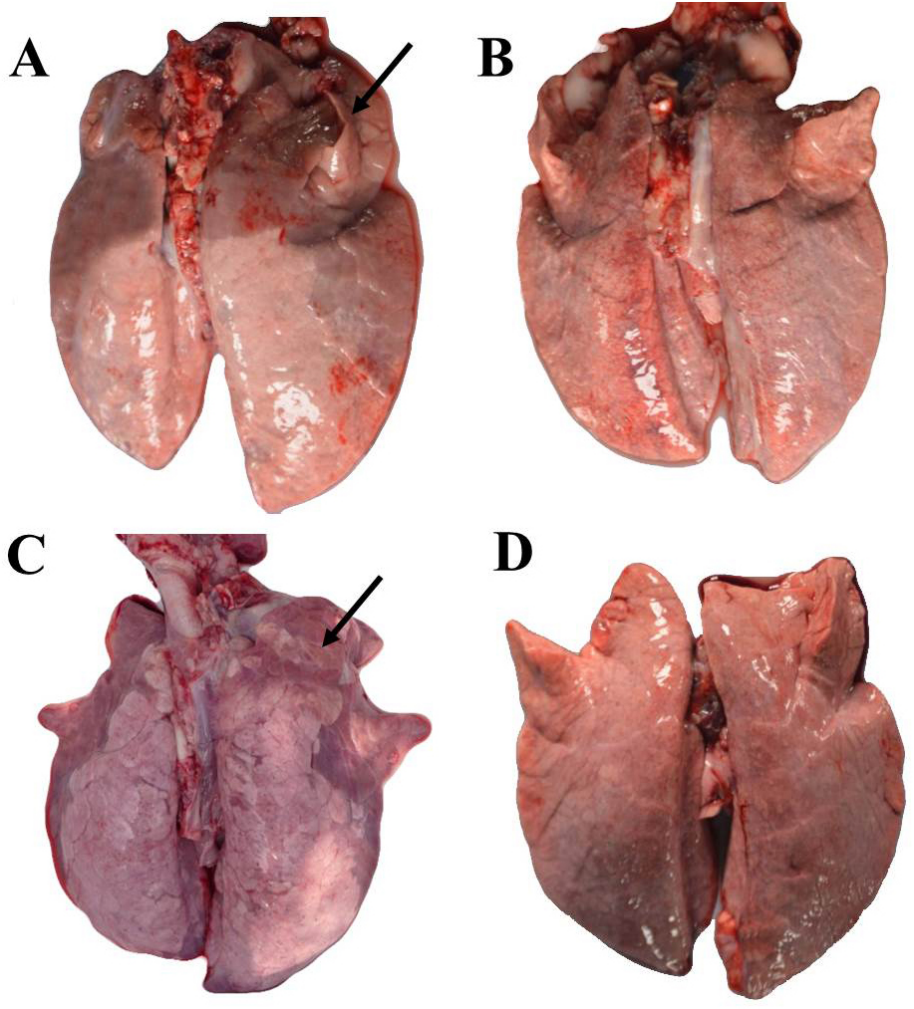
Lung tissue of experimental pigs. (A) Jiangquhai infected pigs, (B) Jiangquhai control pigs, (C) Duroc infected pigs, (D) Duroc control pigs. Lung pathological tissues are indicated with arrows.

**Figure 2:**
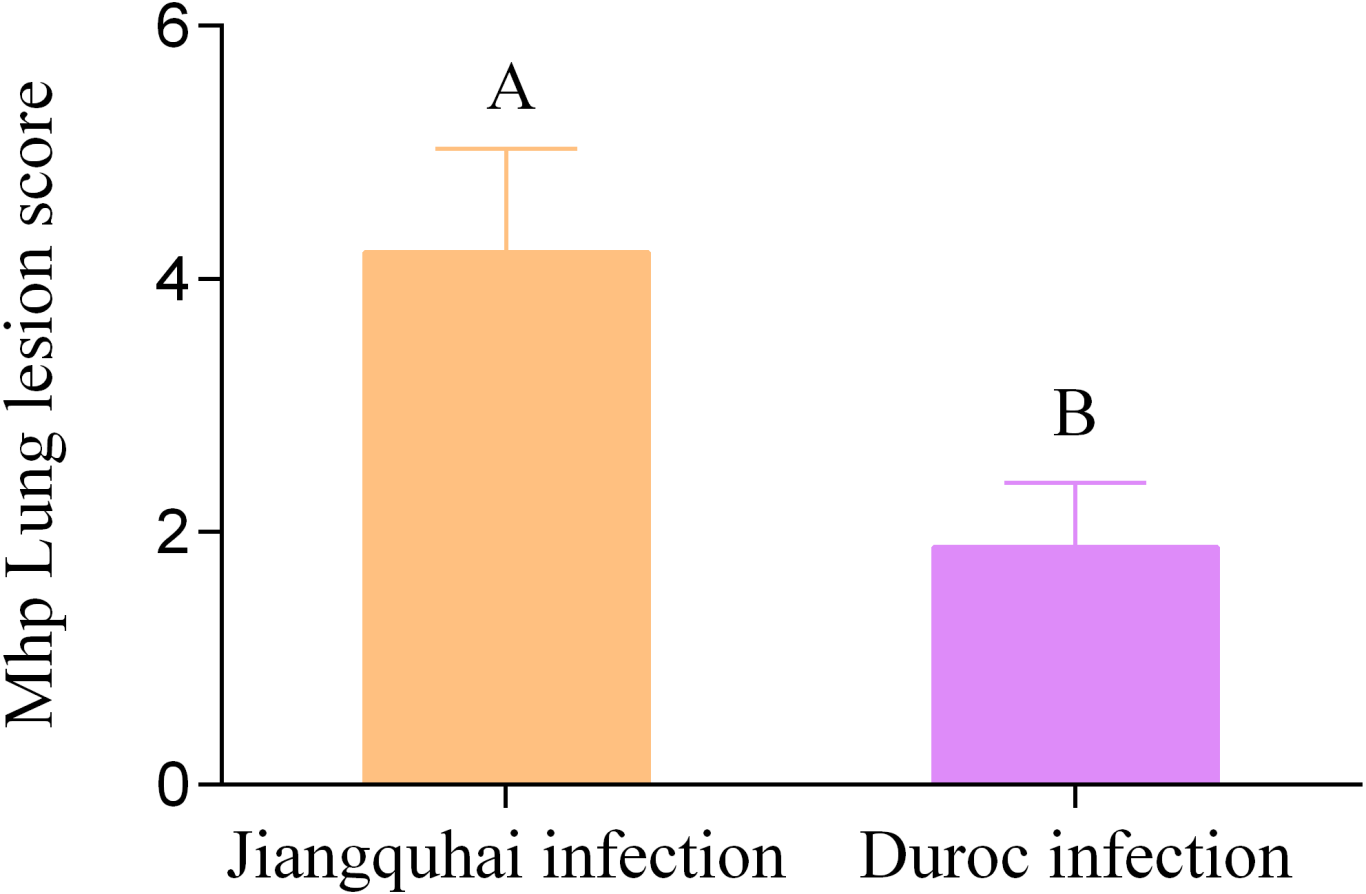
Lung lesion scores. Presence of pulmonary lesions was determined in Jiangquhai infected pigs and Duroc infected pigs. Different letters indicate significant differences (A and B) (*p* < 0.01). Error bars indicate the standard error.

Our analysis of lung tissue on day 28 revealed that Jiangquhai infected pigs had more serious Mhp lung lesions (Fig. 1), a greater difference in Mhp lung lesion scores from Jiangquhai infected pigs compared to Duroc infected pigs (*p* <  0.01) (Fig. 2).

### Preliminary analysis and summary of RNA-Seq data quality

We performed RNA-seq to analyze the transcriptional profile of lung tissue from the four experimental groups. We found that 87.54–96.47% of clean reads were mapped to the reference genome (*Sus scrofa* 11.1), and 80.19–93.33% were uniquely mapped (Table S2). The sample to sample distance heat map showed a good degree of similarity between all three replicates (Fig. S1), indicating that there were no significant differences in gene expression among the biological replicates. These results showed that the RNA-seq data were reliable and met the conditions for differential expression analysis.

### DEGs analysis and RT-qPCR validation

**Figure 3:**
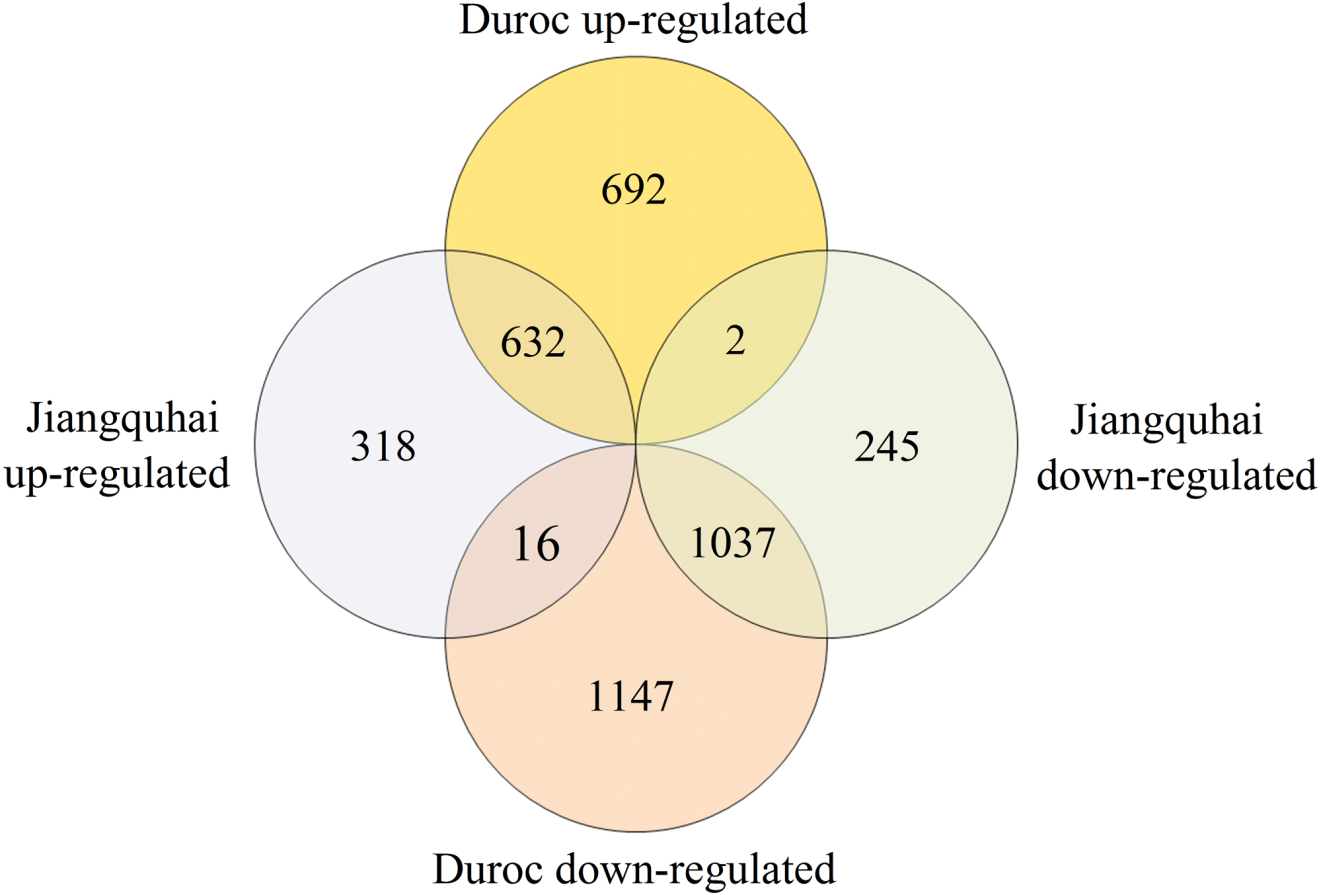
Venn diagram displaying the numbers of DEGs in the two groups. DEGs in infected Jiangquhai pigs and infected Duroc pigs were compared against their control groups. The numbers in the overlapping areas represent DEGs shared between the two groups.

We analyzed the DEG profiles of lung tissues from Mhp-infected Jiangquhai and Duroc pigs by comparing infected and control animals of the same breed. Genes with relative expression levels that showed log_2_ FC >  1 (*p* <  0.05) were considered up-regulated, and those with log_2_ FC <−1 (*p* <  0.05) were considered down-regulated. Of the 2,250 DEGs detected in Jiangquhai pigs, 966 genes were up-regulated and 1,284 down-regulated. Of the 3,526 DEGs detected in Duroc pigs, 1,326 and 2,200 were up- and down-regulated, respectively (Fig. 3). Jiangquhai and Duroc pigs shared 1,669 DEGs in response to Mhp infection, with 632 up-regulated genes and 1,037 down-regulated genes. Further analysis showed that Duroc pigs had 694 uniquely up-regulated DEGs, which was two times higher than the number in Jiangquhai pigs (334 DEGs) (Fig. 3).

**Figure 4:**
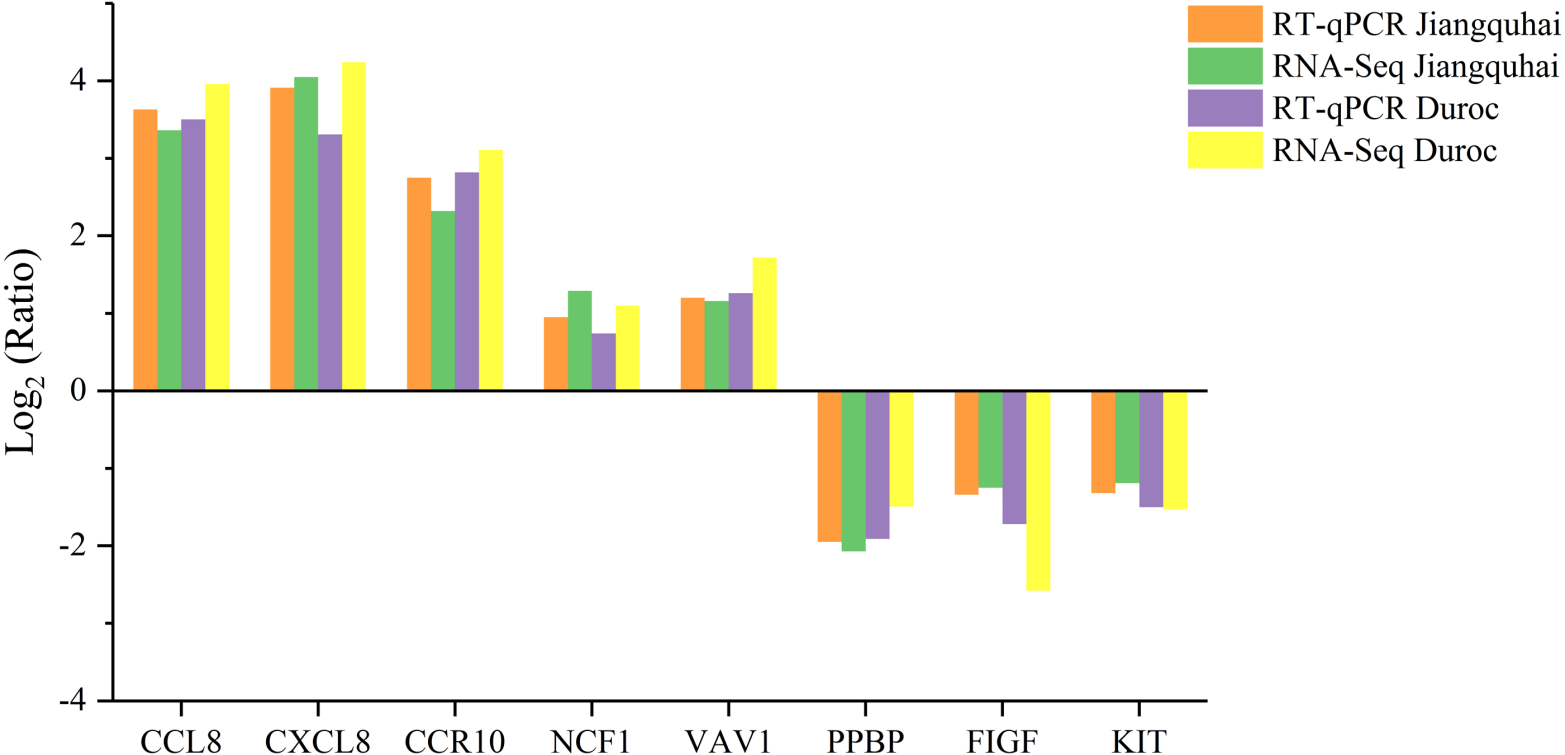
The RT-qPCR identification of randomly selected DEGs and correlation with RNA-seq data. The *X*-axis is the name of genes and the *Y*-axis is the log_2_ Ratio (Treatmented group/Control group) relative expression value.

To verify the RNA-seq results, eight genes were randomly selected for RT-qPCR analysis. Expression FCs determined by RT-qPCR analysis was compared against the profiles predicted by RNA-seq. The RT-qPCR results verified the changes in expression levels of the eight genes (Fig. 4), indicating that the RNA-seq data were reliable.

### Terms and pathways associated with immunobiology were enriched in both breeds

**Figure 5:**
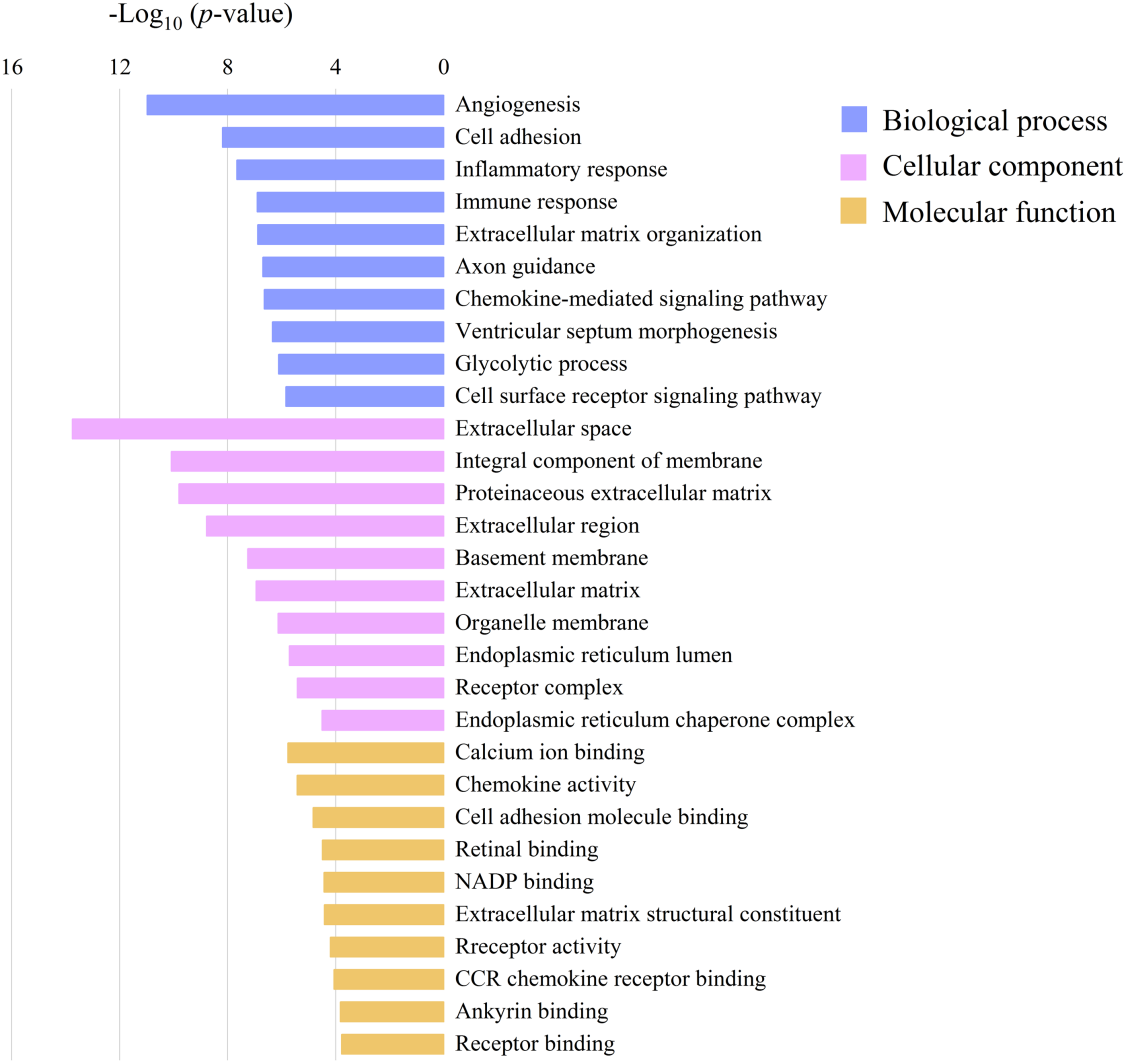
The top 10 gene ontology (GO) enrichments of common DEGs shared by Jiangquhai and Duroc pigs. The *Y*-axis is the name of each category, the *X*-axis is their –log_10_ (*p*-value).

**Figure 6:**
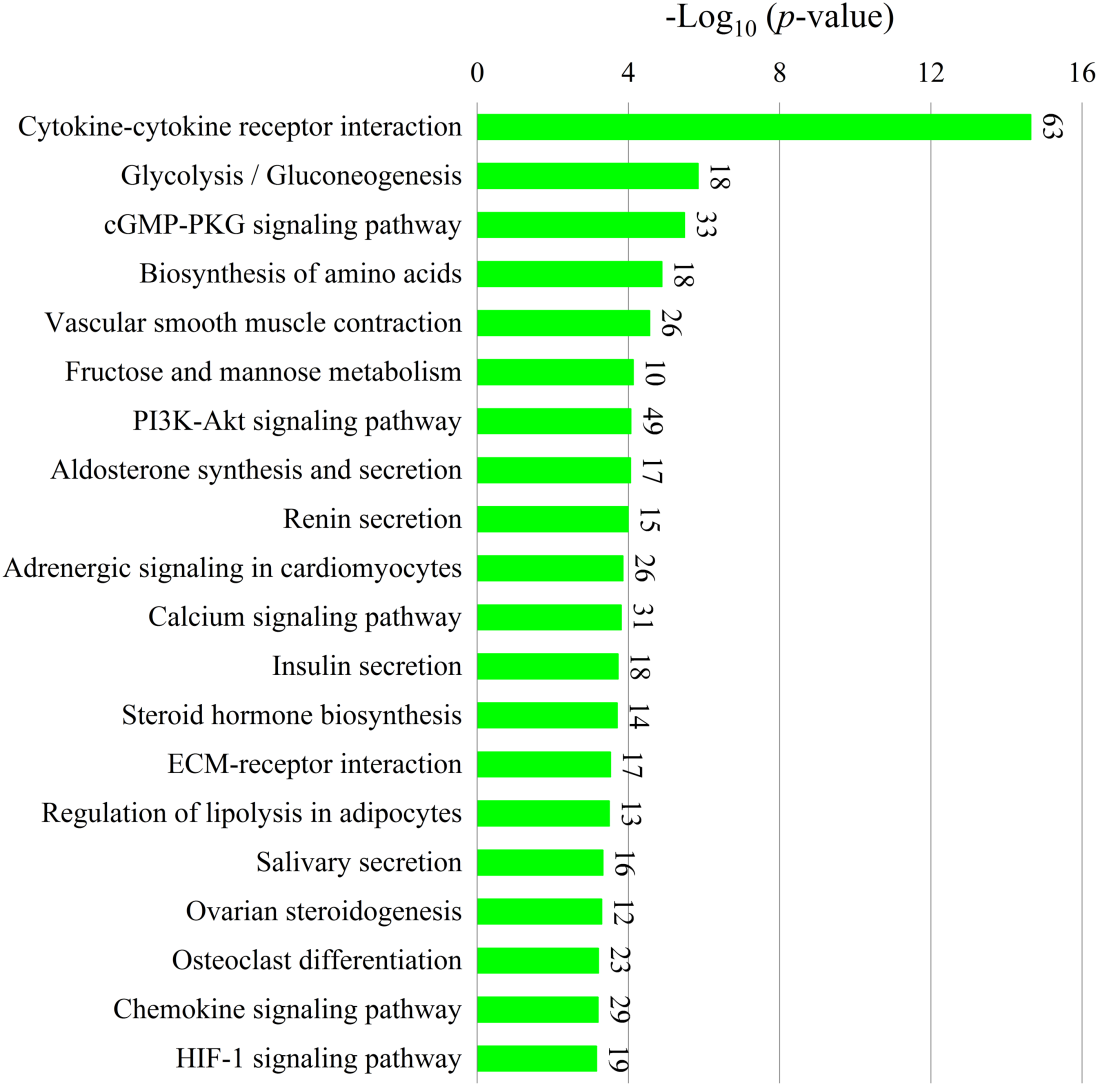
The top 20 Kyoto Encyclopedia of Genes and Genomes (KEGG) pathways of common DEGs shared by Jiangquhai and Duroc pigs. The *Y*-axis is the name of each category, the *X*-axis is their –log_10_ (*p*-value). The number of genes enriched in each category were shown at the top of each bar.

To determine the biological function of DEGs after Mhp infection in lung tissue, the common 1,669 DEGs were submitted to DAVID for GO analysis, which revealed enrichment of 281 GO terms (*p* <  0.05). The top 10 significant enrichments are shown in Fig. 5, among which cell adhesion, inflammatory response and immune response were the most significantly regulated by Mhp infection. Pathway analysis based on the KEGG database revealed the top 20 significant signaling pathways of the two breeds (Fig. 6). Many immune-related pathways were enriched, including the cytokine-cytokine receptor interaction (*IL18*, *IL1R1*), PI3K-Akt signaling pathway (*TLR2*, *ITGA1*, *ITGB7, PIK3R5*), and chemokine signaling pathway (*CXCL8*, *CXCL13*).

### Specific terms and pathways in Jiangquhai and Duroc pigs

Jiangquhai and Duroc pigs also had different responses to Mhp infection. The specific DEGs of Jiangquhai (581 DEGs) and Duroc pigs (1,857 DEGs) were separately submitted to DAVID for GO analysis. The results revealed 100 GO enrichments in Jiangquhai pigs and 314 GO enrichments in Duroc pigs. Table S3 shows the top 10 significant enrichments in Jiangquhai pigs, including cell adhesion, leukocyte migration and extracellular matrix disassembly. Table S4 lists the top 10 significant enrichments in Duroc pigs, including cilium assembly, cilium-dependent cell motility and interleukin-7-mediated signaling pathway.

KEGG pathway analysis revealed 38 specific signaling pathways in Jiangquhai pigs (Table S5) and 54 specific signaling pathways in Duroc pigs (Table S6). Further analysis found that Duroc pigs had more immune-related pathways (18 immune-related pathways), including chemokine signaling pathway, NOD-like receptor signaling pathway, antigen processing and presentation, toll-like receptor signaling pathway, 253 DEGs were enriched in those 18 pathways (Table 2). Jiangquhai pigs only identified 8 immune-related pathways, including leukocyte transendothelial migration, hematopoietic cell lineage, cell adhesion molecules, 99 DEGs were enriched in those 8 pathways (Table 2).

**Table 2:** Important immune-related KEGG pathways of specific DEGs in Jiangquhai pigs and Duroc pigs.

**Breeds**	**Pathway terms**	Categories	**Genes**
**Duroc pigs**	Cytosolic DNA-sensing pathway	Immune system	*IFNB1, NFKBIA, CXCL10, IRF7, ZBP1, CCL5, CCL4, DDX58, IL6, RIPK3, POLR3D, NFKBIB*
	Intestinal immune network for IgA production	Immune system	*CCR9, CXCL12, ICOS, SLA-DRB1, CD28, LOC106504372, IL10, IL6, AICDA, IL15RA*
	Complement and coagulation cascades	Immune system	*C1QA, C1S, F7, BDKRB1, F2, THBD, VSIG4, C1QB, C1QC, VWF, SERPINE1, C7, CPB2, C1R*
	Chemokine signaling pathway	Immune system	*CCR5, CCR1, XCR1, CXCR6, CCR9, CXCL2, NFKBIA, CXCL10, CXCL12, CCL5, AKT3, CCL4, PTK2B, ADCY4, GRK5, JAK3, CXCR3, NFKBIB, LOC110255211, GNG3, PARD3, CXCR2, HCK, ADCY1, PLCB2*
	NOD-like receptor signaling pathway	Immune system	*NFKBIA, CCL5, PSTPIP1, TNFAIP3, IL6, MAPK10, TRIP6, NFKBIB, BIRC3, MAPK11*
	RIG-I-like receptor signaling pathway	Immune system	*IFNB1, NFKBIA, CXCL10, IRF7, ISG15, DHX58, DDX58, TKFC, MAPK10, NFKBIB, TANK, MAPK11*
	Antigen processing and presentation	Immune system	*TAP1, SLA-DRB1, CALR, PDIA3, LOC106504372, CD8B, HSP70.2, IFNG, PSME2, KLRD1, LOC100523789*
	Toll-like receptor signaling pathway	Immune system	*IFNB1, NFKBIA, CXCL10, IRF7, CD14, LY96, CCL5, IFNAR2, AKT3, CCL4, IL6, MAPK10, MAP3K8, MAP2K6, MAPK11*
	T cell receptor signaling pathway	Immune system	*NFKBIA, ICOS, PDCD1, AKT3, CD28, CD8B, IFNG, IL10, CTLA4, NFKBIB, MAP3K8, CARD11, MAPK11, PAK6, NFKBIE*
	TNF signaling pathway	Signal transduction	*CXCL2, NFKBIA, CXCL10, CREB3L4, CCL5, AKT3, TNFAIP3, FAS, PTGS2, IL6, LTA, RIPK3, MAPK10, MAP3K8, MAP2K6, BIRC3, TRAF1, MAPK11*
	Jak-STAT signaling pathway	Signal transduction	*IFNB1, IL7R, SOCS1, IFNAR2, IL27RA, AKT3, IL2RA, CSF3, IFNG, IL10, IL2RG, IL6, JAK3, OSMR, IL15RA, IL20RB, LOC100736818, PIM1, CDKN1A, IL2RB, TSLP, IL22, PTPN2*
	cAMP signaling pathway	Signal transduction	*NFKBIA, TNNI3, CREB3L4, GABBR1, SSTR1, PLD1, AKT3, HTR1B, AMH, ADCY4, MAPK10, GRIA4, GRIA3, PDE4D, SUCNR1, LOC110255845, GRIN3A, PPP1R1B, HCAR2, GRIA1, ADCY1, GLI3, PDE3B, GRIN2A, GRIN2B, CACNA1C, GIPR, CNGB1*
	NF-kappa B signaling pathway	Signal transduction	*NFKBIA, CXCL12, CD14, LY96, BCL2A1, TNFAIP3, CCL4, DDX58, PTGS2, LTA, BIRC3, TRAF1, NFKB2, CARD11*
	MAPK signaling pathway	Signal transduction	*CD14, NTF3, DDIT3, PLA2G4B, RPS6KA1, AKT3, HSP70.2, FAS, EGF, FGF14, NTRK1, FGFR4, MAPK10, DUSP4, PRKCG, GADD45B, MAP3K8, MAP2K6, PTPN7, CACNA2D2, CACNA2D3, FGF12, NFKB2, CACNA1H, DUSP2, MAPK8IP2, MAPK11, CACNA1C, CACNA1I, MAP4K1, CACNA1E*
	FoxO signaling pathway	Signal transduction	*CCNB2, IL7R, PCK2, CCNB1, AKT3, EGF, IL10, IL6, MAPK10, PRKAG2, GADD45B, CCNB3, CDKN1A, IRS2, PLK1, MAPK11*
	Cytokine-cytokine receptor interaction	Signaling molecules and interaction	*CCR5, CCR1, XCR1, CXCR6, CCR9, CXCL2, IFNB1, CXCL10, CXCL12, CCL5, IL7R, IFNAR2, TNFRSF9, CD27, CCL4, IL2RA, FAS, CSF3, IFNG, EGF, IL10, IL2RG, AMH, IL6, LTA, OSMR, CXCR3, IL15RA, IL20RB, LOC100736818, IL18RAP, IL25, IL2RB, CXCR2, TSLP, IL22*
	Cell adhesion molecules (CAMs)	Signaling molecules and interaction	*ICOS, SELL, SLA-DRB1, OCLN, PDCD1, VCAN, CLDN1, CD28, LOC106504372, CD8B, CTLA4, MPZ, L1CAM, TIGIT, NLGN3, LOC100525346, ITGA8, NCAM2, NEGR1*
	Neuroactive ligand–receptor interaction	Signaling molecules and interaction	*P2RY2, BDKRB1, F2, GABBR1, GZMA, CRHR2, SSTR1, P2RY6, TRH, TSPO, HRH4, OPRL1, HTR1B, PTH1R, TAAR1, LTB4R2, THRB, PTGDR, GRIA4, GRIA3, P2RX3, PTGIR, GRIK1, GABRB2, CNR1, LRRC74B, PARD3, GRIN3A, P2RX1, GRM2, GABRR3, GRIK2, GRIA1, GRIN2A, GRIN2B, GIPR*
**Jiangquhai pigs**	Complement and coagulation cascades	Immune system	*C5, MBL1, C6, PLAU, F5, SERPINA5, ITGAM, KNG1, SERPINF2*
	Leukocyte transendothelial migration	Immune system	*CLDN23, CLDN18, CLDN5, PECAM1, ITGAM, TXK, ESAM, GNAI1, PLCG2*
	Natural killer cell mediated cytotoxicity	Immune system	*NCR1, IFNAR1, KLRK1, CD244, LOC100739080, SHC3, LOC100523789, PLCG2*
	Hematopoietic cell lineage	Immune system	*CD4, GP1BB, CD34, CD19, ITGAM, CD7*
	Hippo signaling pathway	Signal transduction	*PARD6B, BMP2, WNT10B, WNT16, GDF7, WNT11, TCF7L1, GDF6, WTIP, FZD1*
	cAMP signaling pathway	Signal transduction	*FXYD2, FOS, PDE4A, DRD2, GNAI1, LOC100738425, LOC110255845, LOC110255846, CACNA1F, PDE4C*
	Notch signaling pathway	Signal transduction	*HES1, DTX2, RBPJL*
	Cell adhesion molecules (CAMs)	Signaling molecules and interaction	*NECTIN1, CD4, CLDN23, CLDN18, CLDN5, PECAM1, CD34, ITGAM, VTCN1, ESAM, CDH4, CNTNAP2*

### Analysis of important DEGs related to immune responses

**Figure 7:**
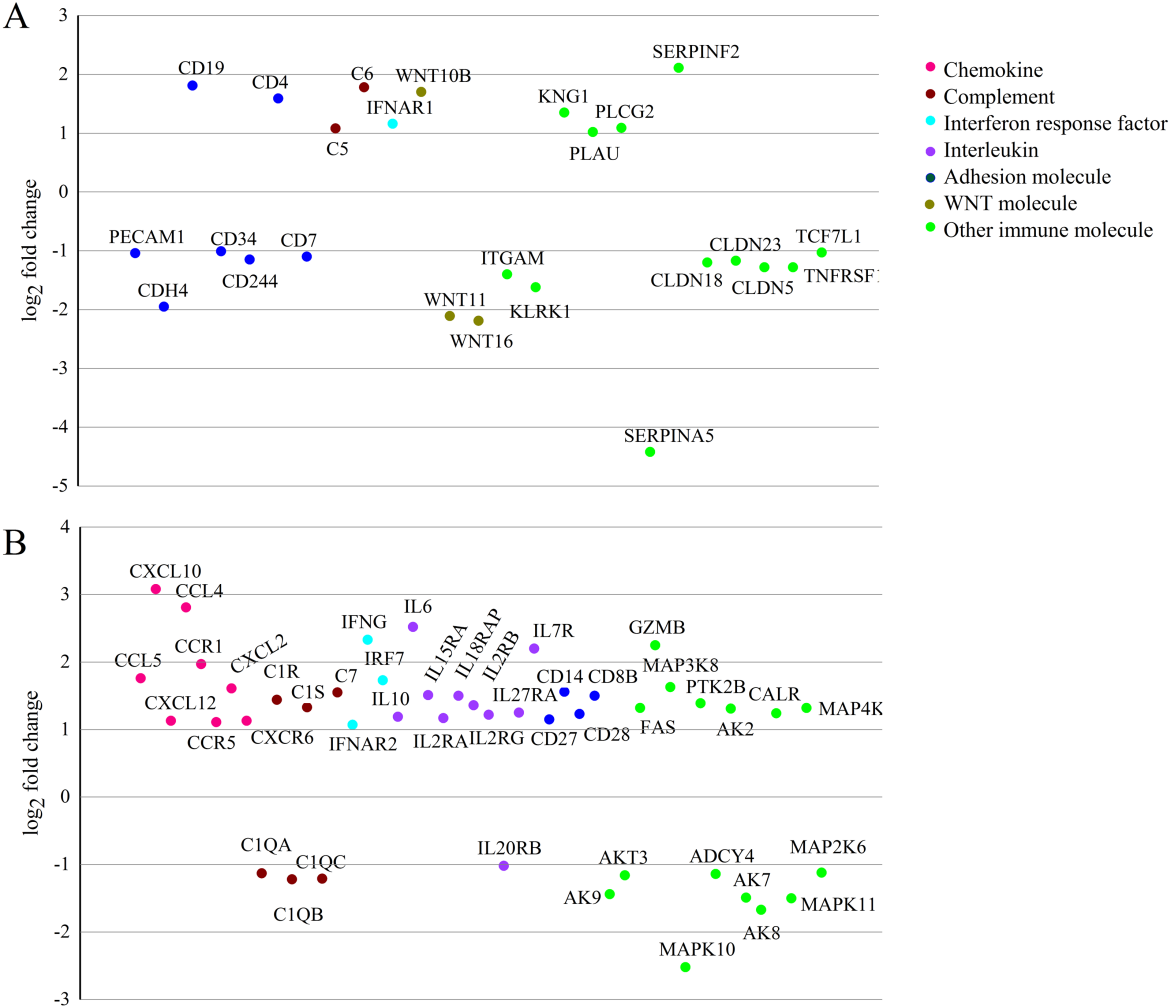
Important immune-related genes showing specific expression in Jiangquhai pigs (A) and Duroc pigs (B). Genes have been arbitrarily positioned along the *x*-axis.

In this study, we mainly focused on the important DEGs related to immune responses. Based on gene clustering and specific KEGG pathways (Table 2), some DEGs that we identified were well-known components of the innate immune response, such as chemokines, interleukins, interferon response factors and complement components. Forty-five important specific DEGs related to innate immune responses had been identified in Duroc pigs (Fig. 7B), and only 20 important specific DEGs had been identified in Jiangquhai pigs (Fig. 7A). Furthermore, some immune-related genes were down-regulated in Jiangquhai pigs, such as adhesion molecules (*PECAM1*, *CD34*, *CD7*, *CDH4*), WNT molecules (*WNT11*, *WNT16*). As for Duroc pigs, more immune-related genes were up-regulated, including chemokines (*CCL4*, *CCL5*, *CCR1*, *CCR5*, *CXCL2*, *CXCL10*, *CXCL12*, *CXCR6*), interferon response factors (*IFNAR2*, *IFNG*), and interleukins (*IL6*, *IL10*, *IL15RA*, *IL2RA*, *IL18RAP*, *IL2RB*, *IL2RG*, *IL27RA*, *IL7R*). Among these immune-related genes, *CXCL10, CCL4*, *IL6* and *IFNG* were the most differently expressed genes.

## Discussion

Previous reports state that the primary effect of Mhp infection on pigs is reduced growth performance (Maes et al., 2008; Pointon, Byrt & Heap, 1985). However, the degree to which Mhp effect differs between Chinese local breeds and imported breeds (such as Duroc and Landrace), and the effect on Chinese local breeds is much more serious (Fang et al., 2015). Our results were consistent with previous reports, Jiangquhai pigs infected with Mhp exhibited poorer growth performance than Duroc pigs. Some studies have reported that Mhp infection causing a humoral immune response in pigs (Blanchard et al., 1992). Moreover, the level of antigen-specific antibody is an important indicator of MPS lesion formation in animals, and increased antigen-specific antibody production may exacerbate MPS lung lesion severity (Borjigin et al., 2016; Davis et al., 1985; Katayama et al., 2011). Our results also showed Mhp-infected Jiangquhai pigs exhibited higher blood levels of Mhp antibody and more serious Mhp lung lesions. These results confirm that Jiangquhai pigs are more susceptible to Mhp infection, and Duroc pigs possess greater resistance. In addition, it was reported that the age and weight of pigs do not affect the susceptibility to Mhp infection (Piffer & Ross, 1984). Therefore, it is theoretically possible that genetic components contribute to Mhp resistance/susceptibility differs among breeds.

To gain insight into how the transcriptome profiles of different pig breeds vary in response to Mhp infection, we performed RNA-seq to analyze the transcriptional profiles of lung tissue from two breeds. We identified 2,250 and 3,526 DEGs in lung tissue from Jiangquhai pigs and Duroc pigs, respectively. Duroc pigs had 694 unique up-regulated DEGs, whereas Jiangquhai pigs only had 334. Taken together, these results indicate that the molecular interactions and signaling pathways following Mhp infection may be more complex in Duroc pigs.

Our analysis revealed 1,669 DEGs shared between Jiangquhai and Duroc pigs in response to Mhp infection. These common DEGs also showed significant enrichment of many immune-relevant terms and pathways. Among these, many up-regulated immune-relevant genes were observed in both breeds that could play a role in resistance to Mhp infection. One TLR family member, *TLR2*, has a fundamental role in pathogen recognition, signal transmission and activation of innate immunity, and stimulation of inflammatory cytokine production (Yoshihiro et al., 2003). *IL18* and *IL1R1* are pro-inflammatory mediators involved in many cytokine-induced immune and inflammatory responses (Dale & Nicklin, 1999). PI3Ks (*PIK3R5*) are important enzymes involved in various signal transduction pathways, and play important roles in regulating cell growth, survival, death and chemotaxis (Margaria et al., 2019). Chemokines such as *CXCL8* and *CXCL13* can recruit immune cells to the site of infection (Strieter et al., 1999; Sun et al., 2006). Moreover, *ITGA1* and *ITGB7*, which help recruit immune cells such as T, B, and natural killer (NK) cells, were up-regulated following Mhp infection (Campbell & Humphries, 2011; Cheli et al., 2007; Lim, Leung & Krissansen, 1998). These immune-related DEGs help clarify the immune response in pigs following Mhp infection.

Comparison of the specific DEGs between Jiangquhai and Duroc pigs revealed some important DEGs related to immune responses were specifically altered in Jiangquhai or Duroc pigs, and these important DEGs enriched in immune-related pathways play an important role in the underlying host mechanism to defend against Mhp infection.

Adhesion molecules are important signal transmitters of the immune system, they function as signal transduction, transported signals from outside to inside of cell (Kumar et al., 2016; Korytina et al., 2019), In this study, many adhesion molecules were specifically down-regulated in Jiangquhai pigs, including *PECAM1*, *CD34*, *CD7* and *CDH4*. These adhesion molecules were significantly enriched in Cell adhesion molecules (CAMs) and Hematopoietic cell lineage pathway, have the function in pathogen recognition, signal transmission and regulating cell movement (Chasiotis et al., 2012; Samanta & Almo, 2015; Xiao et al., 2010). However, these adhesion molecules were down-regulated may reduce host’s capacity for antigen presentation and processing. In addition, two WNT molecules (*WNT11*, *WNT16*) enriched in Hippo signaling pathway were identified in Jiangquhai pigs. WNT genes exert immune modulatory functions during pathogens infection (Brandenburg & Reiling, 2016), and could regulate the expression of immune response genes during challenge by pathogens, such as interferon genes, Toll-like receptors and MHC genes (Garcia-Rodriguez et al., 2017). *WNT11* and *WNT16* were down-regulated, which may induce the inhibition of its downstream signaling, including cell growth, survival and cell-cycle progression. Therefore, thus results may interfere with Jiangquhai pigs to establish an effective immune response against Mhp infection.

Chemokines constitute a large family of chemotactic molecules that are fundamentally involved in the inflammatory response by attracting immune cells to sites of inflammation, and promote the immune response and wound healing (Le et al., 2004; Zlotnik & Yoshie, 2012). Many chemokines are expressed in immune tissues and cells in pigs infected with Mhp (Li et al., 2014; Zhang et al., 2011). In this study, many specific up-regulated chemokines (*CCL4*, *CCL5*, *CCR1*, *CCR5*, *CXCL2*, *CXCL10*, *CXCL12* and *CXCR6*) were identified in Duroc pigs, these chemokines were mainly enriched in cytokine-cytokine receptor interaction and chemokine signaling pathway. The two pathways are involved in specific functional tasks that recruit immune cells to induce inflammatory and adaptive immune responses (Carvalho et al., 2012; Hu et al., 2016). Therefore, these up-regulated chemokines could recruit more immune cells for pathogen defense, and Duroc pigs exhibited a larger chemotactic immune cell capacity. Among these chemokines, *CXCL10 and CCL4* were most highly expressed. *CXCL10* is an important regulator of pulmonary diseases (Gao et al., 2018; Tighe et al., 2011), and has the function of chemotactic monocytes/macrophages, T cells, and NK cells and promotion of T-cell adhesion to endothelial cells (Angiolillo et al., 1995; Dufour et al., 2002). *CCL4* is an important chemoattractant for natural killer cells, monocytes and a variety of other immune cells, regulates immune response to pathogen infection (Bystry et al., 2001; Zhao et al., 2007). Therefore, *CXCL10* and *CCL4* maybe important chemokines involved in chemokine signaling pathway, and regulate the process of immune response to Mhp infection.

Mhp stimulates host immune response by inducing macrophages to release pro-inflammatory cytokines, such as *IL-2*, *IL-6*, *IL-8*, *IL10* and *IL-1β* (Fourour et al., 2019; Lorenzo et al., 2006). These pro-inflammatory cytokines are important players in innate and adaptive immunity, and affect immune balance by suppressing cell-mediated immunity (Zhang et al., 2018). In this study, we also identified some specific up-regulated interleukins (*IL6*, *IL10*, *IL15R A*, *IL2RA*, *IL18RAP*, *IL2RB*, *IL2RG*, *IL27RA* and *IL7R*) in Duroc pigs. These interleukin cytokines can modulate a broad spectrum of immune response processes, such as NOD-like receptor signaling pathway, toll-like receptor signaling pathway, TNF signaling pathway and Jak-STAT signaling pathway. Thus, activation of interleukin cytokines in Duroc pigs may induce additional immune cytokine production and immune cell recruitment for pathogen defense. In addition, interferon response factors have the function of activate macrophages, protect host cells to resist pathogen infection (Rodriguez et al., 2007). Two interferon response factors (*IFNAR2*, *IFNG*) were highly expressed in Duroc pigs, which may further activated macrophages and enhanced the ability to resistance Mhp infection. Moreover, *IL6* and *IFNG* were most highly expressed among these cytokines. *IL-6* is a primary cytokine, which could activate macrophages to secrete inflammatory cytokines and chemokines (Yan et al., 2012), and responsible for Mhp clearance in lungs (Wu et al., 2008). *IFNG* had been detected in the lungs of pathogen-infected pigs and be postulated to be a necessary component for host control of pathogen (Zhang et al., 2011). Therefore, *IL6* and *IFNG* were also important cytokines involved in the regulation of immune response to Mhp infection.

Apoptosis is a mechanism of programmed cell death and is essential for the regulation of immune responses (Rantong & Gunawardena, 2015). Our results further revealed that the apoptosis-related gene (*FAS*) was specifically activated in Duroc pigs. The *FAS* gene has been reported to play a central role in the physiological regulation of programmed cell death and has been implicated in the pathogenesis of various diseases of the immune system (Strasser, Jost & Nagata, 2009). Previous studies have shown that the *FAS* gene involved in the regulation of inflammatory response in pulmonary (Liu et al., 2017). Therefore, the *FAS* gene activation can induce apoptosis and regulate the immune response in response to the Mhp infection. In addition, in the Duroc-specific DEGs involved in GO enrichments (cilium assembly, cilium-dependent cell motility), we found intraflagellar transport (*IFT*) genes, which can modulate primary cilia formation and function (Pedersen & Rosenbaum, 2008), such as *IFT172* and *IFT81*. Primary cilia are found on the cell surface of almost every cell type, which play an important role in signaling and development (Singla & Reiter, 2006) and influence immune cell migration (Finetti, Onnis & Baldari, 2015). Therefore, intraflagellar transport genes may play an important role in the immune response to Mhp infection. Further elucidating the function remains to be a near future goal.

## Conclusions

This is the first study to describe the transcriptional profiles of lung tissue from different pig breeds following Mhp infection. RNA-seq analysis identified 966 up-regulated and 1,284 down-regulated genes in Jiangquhai pigs compared to 1,326 up-regulated and 2,200 down-regulated genes in Duroc pigs. Both breeds shared some KEGG pathways, including cytokine-cytokine receptor interaction, PI3K-Akt signaling pathway, and chemokine signaling pathway. All of these may play important roles in Mhp infection resistance. In Duroc pigs, 1857 specific DEGs were identified, KEGG pathway analysis revealed 18 immune-related pathways. I Jiangquhai pigs, 581 specific DEGs were identified and eight immune-related pathways were identified. Compared to Jiangquhai pigs, chemokines, interferon response factors, interleukins, complement components, apoptosis-related molecule and other immune-related molecules were specifically activated in Duroc pigs, and they may help host enhance immune response and reduce Mhp susceptibility. The results of our analysis reveal an important role of genetics in the immune response to Mhp infection, and this should be investigated further to improve pig health during breeding.

##  Supplemental Information

10.7717/peerj.7900/supp-1 Figure S1 Sample clustering based on RNA-seq profiles Variance-stabilizing transformed count data was used for all samples. The heat map shows a greyscale false colour representation of the Euclidean distance matrix, and the dendrogram represents a hierarchical clustering.

10.7717/peerj.7900/supp-2 Table S1 qRT-PCR Primers

10.7717/peerj.7900/supp-3 Table S2 Alignment statistics of reads align to reference gene

10.7717/peerj.7900/supp-4 Table S3 The top 10 GO enrichments of specific DEGs in Jiangquhai pigs

10.7717/peerj.7900/supp-5 Table S4 The top 10 GO enrichments of specific DEGs in Duroc pigs

10.7717/peerj.7900/supp-6 Table S5 The KEGG Pathways of specific DEGs in Jiangquhai pigs

10.7717/peerj.7900/supp-7 Table S6 The KEGG Pathways of specific DEGs in Duroc pigs

10.7717/peerj.7900/supp-8 Dataset S1 Raw data for Body weight and Mhp-specific antibody levels of experimental pigs (Table 1)

10.7717/peerj.7900/supp-9 Dataset S2 Raw data for Lung lesion score (Fig. 2)

10.7717/peerj.7900/supp-10 Dataset S3 Raw data for the RT-qPCR of randomly selected DEGs (Fig. 4)

## Additional Information and Declarations

### DNA Deposition

The following information was supplied regarding the deposition of DNA sequences: The sequences are available at the NCBI Short Read Archive (SRA) under BioProject: PRJNA542576. Raw reads are available in the Sequence Read Archive database (SRR9114668 –SRR9114679).

### Data Availability

The following information was supplied regarding data availability: The raw measurements are available in Datasets S1–S3.
