# Supplementary figures and images for: RNA-seq transcriptome profiling of porcine lung from two pig breeds in response to Mycoplasma hyopneumoniae infection
Source: PeerJ. 2019 Oct 21;7:e7900. doi: 10.7717/peerj.7900 (PMC6812673; doi:10.7717/peerj.7900)

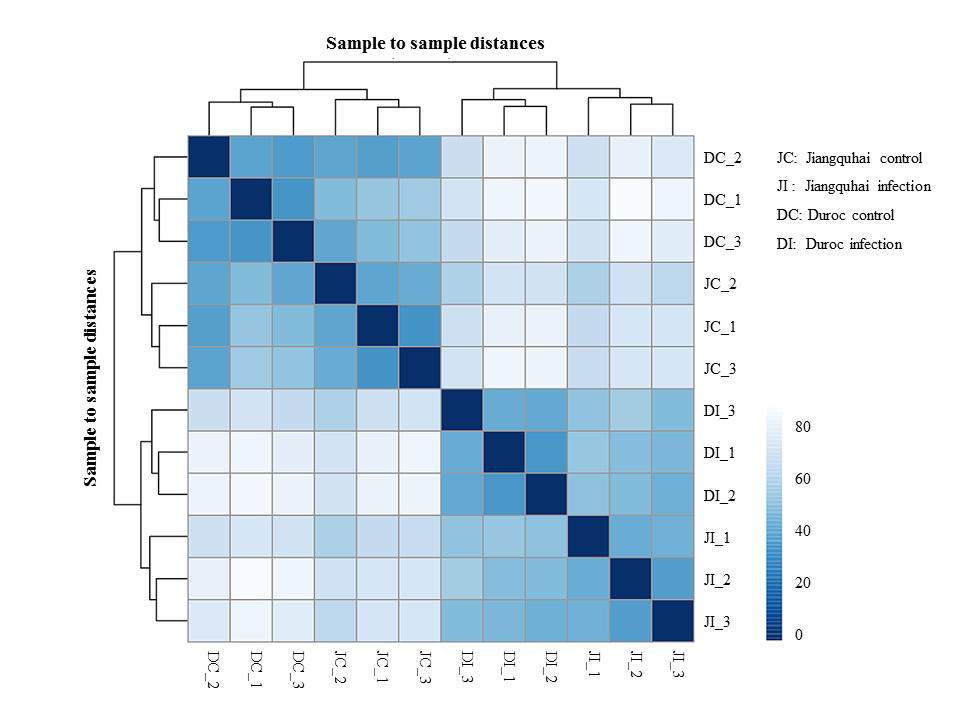

Supplement: Figure S1 — Variance-stabilizing transformed count data was used for all samples. The heat map shows a greyscale false colour representation of the Euclidean distance matrix, and the dendrogram represents a hierarchical clustering. [file peerj-07-7900-s001.png]
