# Supplementary material for: RNA-seq transcriptome profiling of porcine lung from two pig breeds in response to Mycoplasma hyopneumoniae infection
Source: PeerJ. 2019 Oct 21;7:e7900. doi: 10.7717/peerj.7900 (PMC6812673; doi:10.7717/peerj.7900)
Supplement: Table S1 [file peerj-07-7900-s002.docx]

**Table S1 qRT-PCR Primers**

| **Gene** | **Genbank Accession** | **Primer Sequences** | **Size(bp)** | **Annealing (℃)** |
| --- | --- | --- | --- | --- |
|  |  |  |  |  |
| *CCL8* | NM_001164515.1 | F:5'-TTCTGTGTCTGCTGCTCACC-3' | 240 | 58 |
|  |  | R:5'-TGGAATTCTGGACCCACTTC-3' |  |  |
| *CXCL8* | NM_213867.1 | F:5'-CTGTGAGGCTGCAGTTCTG-3' | 206 | 58 |
|  |  | R:5'-CCTTCTGCACCCACTTTTCC-3' |  |  |
| *CCR10* | NM_001044563.1 | F:5'-CTGCCTGCTCTCCTTTTCAG-3' | 266 | 59 |
|  |  | R:5'-GGCAGCTGCAGTATCACAAA-3' |  |  |
| *NCF1* | NM_001113220.1 | F:5'-ATGAGCCTGCCTGTCAAGAT-3' | 262 | 58 |
|  |  | R:5'-TCTTCTCCACGACATCCACC-3' |  |  |
| *VAV1* | NM_001267833.1 | F:5'-CCAGATGTCCCAGTTCCTGT-3' | 225 | 59 |
|  |  | R:5'-TCATCACCCACACTGTCCTC-3' |  |  |
| *PPBP* | NM_213862.2 | F:5'-CTCCAGGTATTGCTGCCATT-3' | 295 | 60 |
|  |  | R:5'-CTGACCCACCATCTTCCATT-3' |  |  |
| *FIGF* | XM_001928382.5 | F:5'-CCATCCAGATCCCAGAAGAA-3' | 289 | 59 |
|  |  | R:5'-CTGACAGCAGCTCTCCACAG-3' |  |  |
| *KIT* | NM_001044525.1 | F:5'-CGTGCTGTCGAAGAAATTCA-3' | 330 | 60 |
|  |  | R:5'-GGTGGTTGTGACATTTGCAG-3' |  |  |
| *GAPDH* | XM_021091114.1 | F:5'-CTTCACACGTGCTGATGGAG-3' | 186 | 60 |
|  |  | R:5'-GGAACATGGGTGAGACCTGT-3' |  |  |
