# Supplementary material for: RNA-seq transcriptome profiling of porcine lung from two pig breeds in response to Mycoplasma hyopneumoniae infection
Source: PeerJ. 2019 Oct 21;7:e7900. doi: 10.7717/peerj.7900 (PMC6812673; doi:10.7717/peerj.7900)
Supplement: Table S2 [file peerj-07-7900-s003.docx]

**Table S2 Alignment statistics of reads align to reference gene**

| **Sample** | **Total reads** | **Total Mapped** | **Uniquely mapped** |
| --- | --- | --- | --- |
| DC 1 | 110071832 | 106189602(96.47%) | 102732429(93.33%) |
| DC 2 | 92161440 | 88038955(95.53%) | 83803604(90.93%) |
| DC 3 | 90938394 | 87311728(96.01%) | 81905749(90.07%) |
| DI 1 | 108085680 | 102098345(94.46%) | 95632053(88.48%) |
| DI 2 | 107102264 | 96134905(89.76%) | 89031661(83.13%) |
| DI 3 | 112810798 | 104789311(92.89%) | 98799057(87.58%) |
| JC 1 | 102811746 | 98262610(95.58%) | 93833817(91.27%) |
| JC 2 | 114858698 | 109041786(94.94%) | 102738251(89.45%) |
| JC 3 | 105488304 | 100491927(95.26%) | 95061490(90.12%) |
| JI 1 | 115186712 | 105477389(91.57%) | 100096146(86.90%) |
| JI 2 | 107118108 | 93771183(87.54%) | 85897829(80.19%) |
| JI 3 | 104030334 | 95546895(91.85%) | 88619031(85.19%) |
