# Supplementary material for: RNA-seq transcriptome profiling of porcine lung from two pig breeds in response to Mycoplasma hyopneumoniae infection
Source: PeerJ. 2019 Oct 21;7:e7900. doi: 10.7717/peerj.7900 (PMC6812673; doi:10.7717/peerj.7900)
Supplement: Table S3 [file peerj-07-7900-s004.docx]

**Table S3. The top 10 GO enrichments of specific DEGs in Jiangquhai pigs**

| **GO ID** | **GO items** | **Category** | **Nunber of DEGs** | ***p*-value** |
| --- | --- | --- | --- | --- |
| GO:1900004 | negative regulation of serine-type endopeptidase activity | Biological process | 4 | 5.69E-07 |
| GO:0006865 | amino acid transport | Biological process | 7 | 2.65E-06 |
| GO:0007155 | cell adhesion | Biological process | 27 | 4.27E-06 |
| GO:0030198 | extracellular matrix organization | Biological process | 12 | 4.91E-05 |
| GO:0030574 | collagen catabolic process | Biological process | 6 | 7.00E-05 |
| GO:0050900 | leukocyte migration | Biological process | 7 | 0.000245 |
| GO:0046718 | viral entry into host cell | Biological process | 4 | 0.000384 |
| GO:0071805 | potassium ion transmembrane transport | Biological process | 7 | 0.000455 |
| GO:0022617 | extracellular matrix disassembly | Biological process | 4 | 0.000484 |
| GO:0003151 | outflow tract morphogenesis | Biological process | 4 | 0.000905 |
| GO:0005615 | extracellular space | Cellular omponent | 66 | 4.95E-11 |
| GO:0005578 | proteinaceous extracellular matrix | Cellular omponent | 28 | 2.22E-10 |
| GO:0005886 | plasma membrane | Cellular omponent | 132 | 9.37E-08 |
| GO:0005576 | extracellular region | Cellular omponent | 53 | 3.09E-05 |
| GO:0005581 | collagen trimer | Cellular omponent | 7 | 0.000358 |
| GO:0070821 | tertiary granule membrane | Cellular omponent | 4 | 0.001092 |
| GO:0016021 | integral component of membrane | Cellular omponent | 126 | 0.001485 |
| GO:0005887 | integral component of plasma membrane | Cellular omponent | 43 | 0.002607 |
| GO:0035579 | specific granule membrane | Cellular omponent | 4 | 0.003295 |
| GO:0005604 | basement membrane | Cellular omponent | 6 | 0.003317 |
| GO:0004867 | serine-type endopeptidase inhibitor activity | Molecular function | 13 | 6.84E-07 |
| GO:0015171 | amino acid transmembrane transporter activity | Molecular function | 8 | 1.45E-06 |
| GO:0015179 | L-amino acid transmembrane transporter activity | Molecular function | 4 | 6.32E-06 |
| GO:0004222 | metalloendopeptidase activity | Molecular function | 11 | 4.18E-05 |
| GO:0005088 | Ras guanyl-nucleotide exchange factor activity | Molecular function | 7 | 6.48E-05 |
| GO:0015189 | L-lysine transmembrane transporter activity | Molecular function | 4 | 9.38E-05 |
| GO:0015181 | arginine transmembrane transporter activity | Molecular function | 4 | 0.000129 |
| GO:0016791 | phosphatase activity | Molecular function | 5 | 0.000149 |
| GO:0004872 | receptor activity | Molecular function | 11 | 0.000419 |
| GO:0030246 | carbohydrate binding | Molecular function | 10 | 0.000847 |
