# Supplementary material for: RNA-seq transcriptome profiling of porcine lung from two pig breeds in response to Mycoplasma hyopneumoniae infection
Source: PeerJ. 2019 Oct 21;7:e7900. doi: 10.7717/peerj.7900 (PMC6812673; doi:10.7717/peerj.7900)
Supplement: Table S4 [file peerj-07-7900-s005.docx]

**Table S4. The top 10 GO enrichments of specific DEGs in Duroc pigs**

| **GO ID** | **GO terms** | **Category** | **Nunber of DEGs** | | ***p*-value** |
| --- | --- | --- | --- | --- | --- |
| GO:0003341 | cilium movement | Biological process | 18 | | 2.97E-12 |
| GO:0060271 | cilium assembly | Biological process | 39 | 2.97E-09 | |
| GO:0006334 | nucleosome assembly | Biological process | 27 | | 5.98E-09 |
| GO:0060285 | cilium-dependent cell motility | Biological process | 8 | | 7.76E-09 |
| GO:0060294 | cilium movement involved in cell motility | Biological process | 7 | | 3.26E-07 |
| GO:0036159 | inner dynein arm assembly | Biological process | 8 | | 4.21E-07 |
| GO:0007018 | microtubule-based movement | Biological process | 19 | | 7.71E-07 |
| GO:0035082 | axoneme assembly | Biological process | 9 | | 8.46E-07 |
| GO:0036158 | outer dynein arm assembly | Biological process | 8 | | 1.08E-06 |
| GO:0038111 | interleukin-7-mediated signaling pathway | Biological process | 7 | | 1.09E-06 |
| GO:0000786 | nucleosome | Cellular component | 36 | | 4.76E-17 |
| GO:0005930 | axoneme | Cellular component | 32 | | 1.24E-16 |
| GO:0031514 | motile cilium | Cellular component | 22 | | 3.19E-08 |
| GO:0005874 | microtubule | Cellular component | 53 | | 9.77E-08 |
| GO:0036126 | sperm flagellum | Cellular component | 12 | | 2.37E-07 |
| GO:0005929 | cilium | Cellular component | 31 | | 9.64E-06 |
| GO:0002177 | manchette | Cellular component | 4 | | 5.07E-05 |
| GO:0005833 | hemoglobin complex | Cellular component | 5 | | 5.83E-05 |
| GO:0005871 | kinesin complex | Cellular component | 12 | | 0.000129 |
| GO:0008250 | oligosaccharyltransferase complex | Cellular component | 5 | | 0.000134 |
| GO:0045503 | dynein light chain binding | Molecular function | 15 | | 3.04E-12 |
| GO:0008017 | microtubule binding | Molecular function | 45 | | 1.33E-09 |
| GO:0008569 | ATP-dependent microtubule motor activity, minus-end-directed | Molecular function | 10 | | 2.23E-08 |
| GO:0003777 | microtubule motor activity | Molecular function | 19 | | 3.71E-08 |
| GO:0045505 | dynein intermediate chain binding | Molecular function | 11 | | 3.82E-07 |
| GO:0045504 | dynein heavy chain binding | Molecular function | 8 | | 4.21E-07 |
| GO:0046982 | protein heterodimerization activity | Molecular function | 59 | | 1.49E-06 |
| GO:0051959 | dynein light intermediate chain binding | Molecular function | 11 | | 2.06E-06 |
| GO:0031492 | nucleosomal DNA binding | Molecular function | 9 | | 6.09E-06 |
| GO:0005234 | extracellularly glutamate-gated ion channel activity | Molecular function | 8 | | 9.91E-06 |
