# Supplementary material for: RNA-seq transcriptome profiling of porcine lung from two pig breeds in response to Mycoplasma hyopneumoniae infection
Source: PeerJ. 2019 Oct 21;7:e7900. doi: 10.7717/peerj.7900 (PMC6812673; doi:10.7717/peerj.7900)
Supplement: Table S5 [file peerj-07-7900-s006.docx]

**Table S5. The KEGG Pathways of specific DEGs in Jiangquhai pigs**

| **Pathway ID** | **Pathway terms** | **Nunber of DEGs** | ***p*-value** |
| --- | --- | --- | --- |
| ko04610 | Complement and coagulation cascades | 9 | 5.77E-05 |
| ko04514 | Cell adhesion molecules (CAMs) | 12 | 0.000212 |
| ko00590 | Arachidonic acid metabolism | 8 | 0.000502 |
| ko00250 | Alanine, aspartate and glutamate metabolism | 5 | 0.000543 |
| ko02010 | ABC transporters | 6 | 0.000639 |
| ko04974 | Protein digestion and absorption | 8 | 0.000786 |
| ko04380 | Osteoclast differentiation | 10 | 0.00141 |
| ko00140 | Steroid hormone biosynthesis | 6 | 0.001547 |
| ko04916 | Melanogenesis | 8 | 0.001878 |
| ko04670 | Leukocyte transendothelial migration | 9 | 0.002283 |
| ko00053 | Ascorbate and aldarate metabolism | 3 | 0.002873 |
| ko04390 | Hippo signaling pathway | 10 | 0.004135 |
| ko04650 | Natural killer cell mediated cytotoxicity | 8 | 0.005651 |
| ko04975 | Fat digestion and absorption | 4 | 0.006477 |
| ko03320 | PPAR signaling pathway | 6 | 0.006535 |
| ko04640 | Hematopoietic cell lineage | 6 | 0.007016 |
| ko00270 | Cysteine and methionine metabolism | 4 | 0.009614 |
| ko00600 | Sphingolipid metabolism | 4 | 0.011518 |
| ko04911 | Insulin secretion | 6 | 0.011849 |
| ko04270 | Vascular smooth muscle contraction | 8 | 0.01242 |
| ko00520 | Amino sugar and nucleotide sugar metabolism | 4 | 0.013664 |
| ko00480 | Glutathione metabolism | 4 | 0.016064 |
| ko00983 | Drug metabolism - other enzymes | 3 | 0.016569 |
| ko01230 | Biosynthesis of amino acids | 5 | 0.017176 |
| ko04972 | Pancreatic secretion | 6 | 0.019752 |
| ko00982 | Drug metabolism - cytochrome P450 | 4 | 0.020167 |
| ko00591 | Linoleic acid metabolism | 3 | 0.024102 |
| ko04970 | Salivary secretion | 5 | 0.02465 |
| ko04976 | Bile secretion | 5 | 0.02465 |
| ko00980 | Metabolism of xenobiotics by cytochrome P450 | 4 | 0.026626 |
| ko04024 | cAMP signaling pathway | 10 | 0.02743 |
| ko04730 | Long-term depression | 4 | 0.028424 |
| ko00260 | Glycine, serine and threonine metabolism | 3 | 0.028531 |
| ko04924 | Renin secretion | 4 | 0.034275 |
| ko00565 | Ether lipid metabolism | 3 | 0.036026 |
| ko00830 | Retinol metabolism | 4 | 0.040822 |
| ko04330 | Notch signaling pathway | 3 | 0.044555 |
| ko00330 | Arginine and proline metabolism | 3 | 0.047629 |
