# Supplementary material for: RNA-seq transcriptome profiling of porcine lung from two pig breeds in response to Mycoplasma hyopneumoniae infection
Source: PeerJ. 2019 Oct 21;7:e7900. doi: 10.7717/peerj.7900 (PMC6812673; doi:10.7717/peerj.7900)
Supplement: Table S6 [file peerj-07-7900-s007.docx]

**Table S6. The KEGG Pathways of specific DEGs in Duroc pigs**

| **Pathway ID** | **Pathway terms** | **Nunber of DEGs** | ***p*-value** |
| --- | --- | --- | --- |
| ko04724 | Glutamatergic synapse | 22 | 3.94E-05 |
| ko04210 | Apoptosis | 24 | 0.000114 |
| ko04060 | Cytokine-cytokine receptor interaction | 37 | 0.00015 |
| ko04713 | Circadian entrainment | 18 | 0.000222 |
| ko04623 | Cytosolic DNA-sensing pathway | 12 | 0.000503 |
| ko04914 | Progesterone-mediated oocyte maturation | 16 | 0.00061 |
| ko04672 | Intestinal immune network for IgA production | 10 | 0.00066 |
| ko04723 | Retrograde endocannabinoid signaling | 18 | 0.000665 |
| ko04728 | Dopaminergic synapse | 20 | 0.000955 |
| ko04668 | TNF signaling pathway | 18 | 0.000966 |
| ko04110 | Cell cycle | 20 | 0.00119 |
| ko04610 | Complement and coagulation cascades | 14 | 0.001258 |
| ko04062 | Chemokine signaling pathway | 26 | 0.001715 |
| ko04630 | Jak-STAT signaling pathway | 23 | 0.001731 |
| ko04024 | cAMP signaling pathway | 28 | 0.001754 |
| ko03030 | DNA replication | 8 | 0.001946 |
| ko04621 | NOD-like receptor signaling pathway | 10 | 0.002101 |
| ko04622 | RIG-I-like receptor signaling pathway | 12 | 0.002605 |
| ko04710 | Circadian rhythm | 7 | 0.003361 |
| ko04111 | Cell cycle - yeast | 12 | 0.003444 |
| ko04612 | Antigen processing and presentation | 11 | 0.003759 |
| ko00230 | Purine metabolism | 24 | 0.00446 |
| ko04115 | p53 signaling pathway | 12 | 0.004491 |
| ko04064 | NF-kappa B signaling pathway | 14 | 0.004519 |
| ko04726 | Serotonergic synapse | 17 | 0.007335 |
| ko04620 | Toll-like receptor signaling pathway | 15 | 0.007448 |
| ko03050 | Proteasome | 8 | 0.00848 |
| ko04141 | Protein processing in endoplasmic reticulum | 22 | 0.008878 |
| ko04010 | MAPK signaling pathway | 31 | 0.009098 |
| ko00670 | One carbon pool by folate | 4 | 0.011038 |
| ko04113 | Meiosis - yeast | 9 | 0.01191 |
| ko00680 | Methane metabolism | 5 | 0.012663 |
| ko04514 | Cell adhesion molecules (CAMs) | 19 | 0.013227 |
| ko04660 | T cell receptor signaling pathway | 15 | 0.014219 |
| ko00920 | Sulfur metabolism | 3 | 0.01433 |
| ko04080 | Neuroactive ligand-receptor interaction | 36 | 0.014668 |
| ko04920 | Adipocytokine signaling pathway | 11 | 0.014724 |
| ko04966 | Collecting duct acid secretion | 5 | 0.01876 |
| ko04215 | Apoptosis - multiple species | 6 | 0.021157 |
| ko00980 | Metabolism of xenobiotics by cytochrome P450 | 9 | 0.021834 |
| ko00360 | Phenylalanine metabolism | 4 | 0.02241 |
| ko04540 | Gap junction | 12 | 0.022788 |
| ko04360 | Axon guidance | 22 | 0.023892 |
| ko04711 | Circadian rhythm - fly | 2 | 0.024235 |
| ko00750 | Vitamin B6 metabolism | 2 | 0.024235 |
| ko04913 | Ovarian steroidogenesis | 8 | 0.025105 |
| ko04114 | Oocyte meiosis | 14 | 0.034153 |
| ko00590 | Arachidonic acid metabolism | 11 | 0.03482 |
| ko04145 | Phagosome | 18 | 0.035022 |
| ko04750 | Inflammatory mediator regulation of TRP channels | 14 | 0.036712 |
| ko04744 | Phototransduction | 4 | 0.039478 |
| ko00340 | Histidine metabolism | 4 | 0.046534 |
| ko04068 | FoxO signaling pathway | 16 | 0.048078 |
| ko00450 | Selenocompound metabolism | 3 | 0.049241 |
